# Ongoing Progress on Pervaporation Membranes for Ethanol Separation

**DOI:** 10.3390/membranes13100848

**Published:** 2023-10-23

**Authors:** Muhammad Imad, Roberto Castro-Muñoz

**Affiliations:** 1 Department of Process and Systems Engineering, Otto-von-Guericke University, 39106 Magdeburg, Germany; 2 Department of Chemical and Energy Engineering, Pak-Austria Fachhochschule, Haripur 22620, Pakistan; 3 Tecnologico de Monterrey, Campus Toluca, Avenida Eduardo Monroy Cárdenas 2000 San Antonio Buenavista, Toluca de Lerdo 50110, Mexico; 4 Department of Sanitary Engineering, Faculty of Civil and Environmental Engineering, Gdansk University of Technology, G. Narutowicza St. 11/12, 80-233 Gdansk, Poland

**Keywords:** molecular separations, emerging materials, membrane process, ethanol upgrading

## Abstract

Ethanol, a versatile chemical extensively employed in several fields, including fuel production, food and beverage, pharmaceutical and healthcare industries, and chemical manufacturing, continues to witness expanding applications. Consequently, there is an ongoing need for cost-effective and environmentally friendly purification technologies for this organic compound in both diluted (ethanol-water–) and concentrated solutions (water-ethanol–). Pervaporation (PV), as a membrane technology, has emerged as a promising solution offering significant reductions in energy and resource consumption during the production of high-purity components. This review aims to provide a panorama of the recent advancements in materials adapted into PV membranes, encompassing polymeric membranes (and possible blending), inorganic membranes, mixed-matrix membranes, and emerging two-dimensional-material membranes. Among these membrane materials, we discuss the ones providing the most relevant performance in separating ethanol from the liquid systems of water–ethanol and ethanol–water, among others. Furthermore, this review identifies the challenges and future opportunities in material design and fabrication techniques, and the establishment of structure–performance relationships. These endeavors aim to propel the development of next-generation pervaporation membranes with an enhanced separation efficiency.

## 1. Introduction

**Figure 1:**
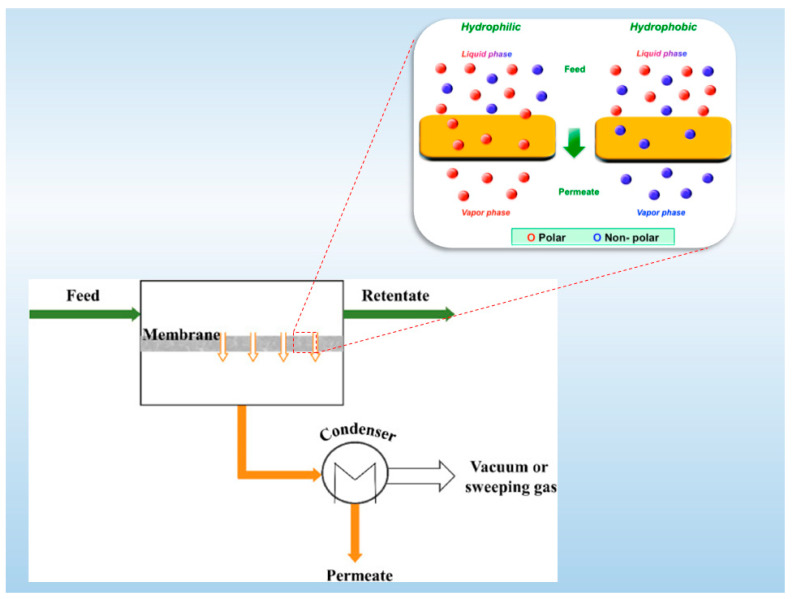
Schematic illustration of the pervaporation separation process [8].

Ethanol, also known as ethyl alcohol, has gained significant attention due to its diverse applications in various industries, including fuel production, food and beverages, pharmaceuticals, healthcare industry, and chemical manufacturing. However, ethanol obtained through the fermentation process typically exhibits a low concentration ranging from 5% to 12% by weight [1]. Consequently, the purification of ethanol from mixtures necessitates a dehydration step [2,3]. Traditional dehydration methods, such as distillation, may face limitations due to the occurrence of an azeotropic point between organic compounds and water. In addition to this, when the organic (ethanol) compound is aimed at being separated directly from the fermentation broth, its separation/extraction is challenging due to the complexity of the media. To overcome these limitations, the pervaporation (PV) process, as illustrated in Figure 1, has emerged as a promising alternative, offering several notable advantages, such as cost-effectiveness, low operating temperatures, a straightforward operational process, and a high production efficiency [4,5,6]. It is important to emphasize that the membrane plays a pivotal role in the PV separation process [7]. Thus, careful consideration of the materials used in membrane preparation, as well as the membrane’s structure, is crucial to enhance the PV separation performance.

**Figure 2:**
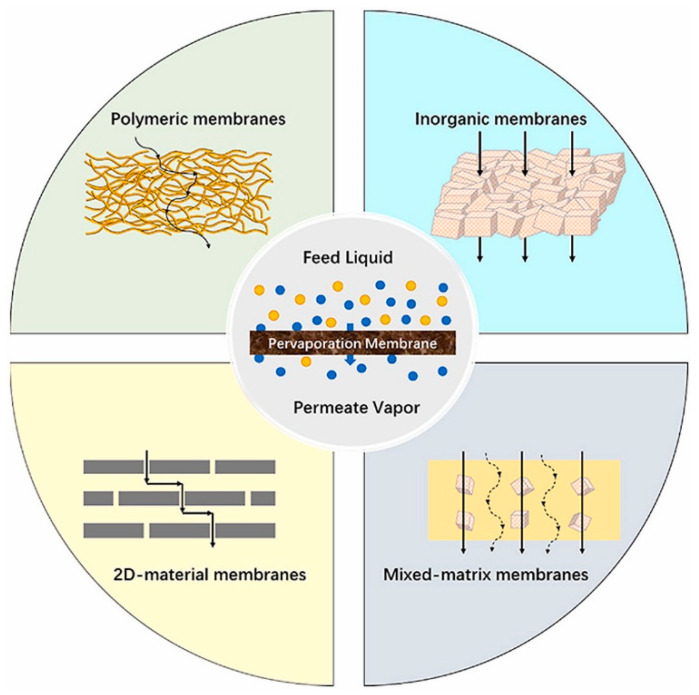
Mechanism of transport channels occurring in various membrane materials in PV technology [25].

In the context of pervaporation, membrane materials are commonly categorized into polymeric membranes, inorganic membranes (including 2D materials membranes) [9], and their synergistic combination such as mixed matrix membranes (MMMs) [10,11] and thin-film composite (TFC) membranes [12]. Figure 2 graphically illustrates these different types of membrane materials employed for PV processes and the hypothesized mechanism of transport across such materials. Polymeric membranes are widely preferred for PV applications owing to their cost-effectiveness, ease of processing, and tunable transport properties [13,14,15,16]. However, the utilization of polymer membranes is constrained by their limited resistance to contamination, low chemical stability, inadequate thermal stability, and the inherent trade-off between permeability and selectivity [17,18]. In contrast, inorganic membranes exhibit notable chemical and thermal stability, along with superior resistance to solvent swelling and excellent mechanical properties [13,19,20,21]. Unfortunately, the inferior film-forming properties and brittleness of inorganic membranes pose challenges for the fabrication of defect-free membranes, consequently limiting their widespread application [13]. In this regard, MMMs, composites, and TFCs present an attractive combination offering various advantages of polymers and inorganic materials [22,23]. However, achieving MMMs with an exceptional performance has proven to be challenging due to the inherent difficulties in achieving uniform filler dispersion and effectively suppressing interfacial voids and a defect-free structure for selective layers [13,17,24].

In recent years, a number of comprehensive reviews have addressed various aspects of pervaporation separation, often focusing on specific types of membrane materials [16,26,27] or particular applications [15,28,29,30]. However, to the best of our knowledge, there has not been a detailed overview of the ongoing and most recent developments in pervaporation (PV) membranes, particularly with a primary emphasis on ethanol dehydration. As ethanol is the leading organic solvent used in many manufacturing industries, PV requires superior materials with high selectivity when adapted into membranes. Therefore, the present review paper offers a comprehensive overview of the ongoing research and recent advancements in the field of PV applied to ethanol dehydration (water–ethanol mixtures) and the simultaneous production and extraction of ethanol (ethanol–water mixtures). Furthermore, it conducts an in-depth analysis of polymer membranes, inorganic membranes, thin-film composites, and mixed matrix materials utilized in this context. Notably, considerable emphasis is placed on the recovery of ethanol from ethanol–water mixtures, accompanied by extensive investigations into the separation performance of developed membranes.

## 2. Polymeric Membranes

Polymeric materials have gained widespread utilization as membrane materials in the chemical industry due to their abundant availability and exceptional film-forming properties. The application of these materials in PV processes for the concentration of organic solvents has witnessed remarkable progress since the 1990s. In recent years, polymeric membranes have emerged as a promising choice for the dehydration of diverse organic mixtures, including water–alcohol (ethanol and isopropanol) [31,32,33], water–acetone [34], and water–ethylene glycol [28,35], among others [32,36,37,38]. The incorporation of thermally stable rigid chain structures within polymer membranes enhances their ability to selectively adsorb target components [3,28], leading to an outstanding dehydration performance for aqueous mixtures of organic liquids [15,32,34]. Of particular significance, hydrophilic polymeric membranes exhibit a notable durability to water, primarily attributed to their functioning as molecular sieves during the dehydration process and their exceptional separation capabilities thanks to their water affinity [28].

Polyvinyl acetate (PVAc), for instance, exhibits significant potential as a polymeric membrane material for industrial separations aimed at concentrating organic solvents, including ethanol [17], acetic acid [15], and isopropanol [2]. This material holds promise due to its notable attributes, including its high chemical stability, good abrasion resistance, high hydrophilicity, and high flexibility. When considering polymeric materials with suitable stability for PV membranes, careful attention must be given to the nature of the cross-linker and the transition temperature, as these factors significantly impact the overall dehydration performance. Specifically, the cross-linker type influences membrane properties and separation capabilities, while the transition temperature determines the degree of membrane brittleness. Consequently, these aspects play a crucial role in achieving a desirable dehydration performance. Recent research efforts have focused on enhancing the separation performance of polymeric membranes for the dehydration of various organic mixtures, demonstrating ongoing endeavors to improve the efficiency and effectiveness of these membranes [39,40,41].

While pure polymeric membranes are widely used for the separation of various organic mixtures, their performance in low pH environments is often suboptimal. These membranes require extensive pretreatment and modifications to withstand acidic conditions. However, within the realm of polymeric materials, polyphenylsulfones have garnered significant attention for their appealing applications in membrane-based technologies, including fuel cell membranes [42,43,44] and industrial separation [45,46]. Notably, these materials have also been investigated for their potential in membrane-based separation technologies for the dehydration of organic mixtures under acidic conditions [43,47]. These studies confirm the immense potential of polymeric membranes, such as polyphenylsulfones, in industrial separation processes, particularly when operating in the presence of an acidic medium.

In principle, polymeric PV membranes can be classified into two categories based on their affinity: hydrophilic and hydrophobic membranes [48,49]. Hydrophilic polymeric membranes exhibit selective permeation of water over organic compounds (with less polarity than water), while hydrophobic polymeric membranes demonstrate selective permeation of organic compounds over water. In the realm of ethanol dehydration (water–ethanol mixtures), a diverse range of hydrophilic polymer membranes have been extensively investigated for PV applications. Some examples of such membranes include poly (vinyl alcohol) (PVA) [17], polyelectrolyte complex (PEC) [50], polyamide (PA) [51], polyimide (PI) [52,53], cellulose [54], chitosan, polyacrylonitrile (PAN) [38], and sodium alginate (SA) [55], among others. As shown in Table 1, these membranes have demonstrated promising potential for the separation of traces of water in ethanol mixtures in PV processes at different operating temperatures. The separation factor can range from 3 up to 2000, depending on the intrinsic properties of the as-prepared polymer membrane.

Compared with hydrophilic PV membranes, the number of hydrophobic membranes identified for this application is relatively limited. This can be attributed to the restricted availability of suitable hydrophobic materials capable of forming appropriate pore structures for molecular separation translated to low permeation. Among the few hydrophobic materials that have demonstrated potential for PVs, notable examples include polydimethylsiloxane (PDMS) and poly(ether-block-amide) (PEBA) [56,57]. Even if these materials have shown the ability to be fabricated into membranes with suitable characteristics for effective molecular separation, further research and development efforts are necessary to expand the repertoire of hydrophobic membranes for enhanced PV performance when directly extracting ethanol molecules.

**Table 1:** PV dehydration of ethanol through various polymeric membranes.

Polymer	Feed (wt.% Ethanol)	Temperature (°C)	Separation Factor	Flux (g/m^2^h)	Ref.
Polyacrylonitrile-polyvinylpyrrolidone	96	20	3.2	2200	[58]
Poly(acrylic acid-co-acrylonitrile)	82	15	877	13	[59]
Poly(vinyl chloride)	96	40	63	3	[60]
Cellulose acetate	96	60	5.9	200	[61]
PVA/25% TEOS, annealed at 160 °C	85	40	329	5	[61]
PVA/25% TEOS, annealed at 130 °C	85	40	893	4	[61]
Chitosan	96	40	2208	4	[62]

### 2.1. Hydrophilic Polymers

The PV dehydration of ethanol utilizes a diverse range of hydrophilic polymer membranes, including poly(vinyl alcohol) (PVA), polyelectrolyte complex (PEC), polyamide (PA), polyimide (PI), cellulose, chitosan, polyacrylonitrile (PAN), and sodium alginate (SA) [63,64,65,66,67,68,69]. Among these membranes, PVA has emerged as a highly investigated and industrially employed option due to its favorable attributes, such as low cost, ease of preparation, and satisfactory performance [25]. However, PVA membranes, while displaying excellent permselectivity for water, typically exhibit relatively low permeation flux (generally less than 300 g/m^2^h) [25]. Additionally, the water solubility of PVA leads to membrane swelling in aqueous media, which can be mitigated through various cross-linking methods; however, this often comes at the expense of reduced permeation flux [17]. Consequently, numerous approaches have been explored to enhance the separation performance of PVA membranes, encompassing cross-linking, filling, and chemical modification techniques [2,17,54].

To some extent, cross-linking of PVA membranes can improve their swelling properties and selectivity; however, it often results in a significant decrease in permeability due to the inherent permeability–selectivity trade-off effect [17]. Overcoming this trade-off behavior has been effectively addressed with mixed matrix membranes (MMMs), which combine inorganic fillers with organic polymer matrices [50,56]. The selection of an appropriate filler material is critical, considering factors such as achieving a high separation performance, either through transport and separation mechanisms (molecular sieving or adsorption), and achieving a good compatibility between filler particles and the polymer matrix [17,64]. Notably, two-dimensional (2D) nanomaterials, such as graphene [35], graphene oxide (GO) [31,64], graphitic carbon nitride (g-C_3_N_4_) [65], and MXene [66], have garnered significant attention regarding the PV dehydration of ethanol due to their unique intrinsic properties, including hydrophilicity and the presence of 2D transport channels within the stacked sheets. Heydari et al., for instance, successfully fabricated PVA/MXene membranes for the PV dehydration of ethanol [17]. The 2D material allowed the composite membranes to exhibit a permeation of ca. 942 g/m^2^h; this means an increase of 106% compared with the bare PVA membranes, while the separation factor was as high as 294, which means enhancements of 79% in comparison with the bare specimens. Comparable selectivity (separation factor of ca. 263) was also reported by Castro-Muñoz et al. [67], who incorporated GO into the PVA matrix followed by chemical cross-linking. Of course, there is evidence of separation performance and mechanical improvement compared with the bare PVA; however, cross-linking limited the permeation properties slightly. In a different work, Wang et al. [68] conducted a study on PVA hybrid membranes containing different types of g-C_3_N_4_ for the PV dehydration of ethanol [4]. The investigation revealed that as the binding force between the polymer and inorganic interface increased, the total flux for the 90 wt.% ethanol–water mixture at 75 °C decreased from 4634 to 2328 g/m^2^ h, while the separation factor increased from 32.4 to 57.9. At this point, more permeable PVA-based membranes were obtained, but with compromised selective properties.

In a separate study, Xia et al. [69] incorporated organosilica into a PVA nanohybrid membrane and evaluated its performance for ethanol dehydration using an 85 vol.% ethanol aqueous solution at 40 °C [69]. The study demonstrated that the PVA–PTES hybrid membrane achieved an optimal performance, with the highest flux recorded at 145 g/m^2^·h and the best separation factor being 1026 [69]. These findings highlight the potential of hybrid membranes, such as PVA-based membranes with g-C_3_N_4_ or organosilica incorporated, to enhance the separation performance in the removal of traces of water from ethanol.

To further enhance the efficiency of the PV process for ethanol dehydration, Gabriela et al. [36], very recently, developed a series of novel PVA membranes filled with chitosan (CS) and various chitosan derivatives microparticles, including phosphorylated chitosan (CS-P), glycidol-modified chitosan (CS-G), glutaraldehyde cross-linked chitosan (CS-GA), and sulphated chitosan (CS-SO3). This study aimed to investigate the impact of these organic fillers on the performance of PVA membranes, as they have demonstrated better effectiveness in ethanol dehydration compared with alginate ones. The newly developed hybrid PVA membranes, composed of a PVA matrix with different chitosan particles (CS, CS-P, CS-GA, CS-G, and CS-SO3), cross-linked with glutaraldehyde, were specifically designed for ethanol dehydration applications. The PV experiments conducted confirmed that the incorporation of various chitosan particles into the PVA matrix had a positive influence on the efficiency of water–ethanol separation [36]. Notably, the membranes containing CS-P and CS-G fillers exhibited the highest fluxes, reaching 1.58 and 1.59 kg/m^2^h, respectively. These observations were well-correlated with the significant enhancement in the hydrophilic properties of the membranes containing these two types of CS derivatives [36]. The findings suggest that the incorporation of chitosan particles, particularly CS-P and CS-G, holds promise for improving the performance of PVA membranes in ethanol dehydration.

To surpass the typical trade-off between membrane selectivity and permeation in the PV of ethanol–water mixtures, Asmaa et al. [70] developed PVA nanocomposite membranes containing different silver concentrations. The synthesized nanocomposite membranes were named M0, M0.5, M1, M1.5, M2, and M2.5, containing AgNO_3_ concentrations of 0 wt.%, 0.5 wt.%, 1 wt.%, 1.5 wt.%, 2 wt.%, and 2.5 wt.%, respectively. The PV tests conducted on the synthesized AgNPs-PVA membranes demonstrated favorable outcomes. Notably, these membranes exhibited high permeability for water while maintaining stable selectivity values, particularly at elevated temperatures and higher ethanol concentrations in the feed [70]. Consequently, the AgNPs-PVA membranes showcased superior performance compared with other PVA-based nanocomposite membranes in terms of the separation of water and ethanol. The results proved that with increasing silver nanoparticle loading, the permeation flux also increased with a drop in separation factor [70]. Specifically, for the M2.5 membrane composition, at a temperature of 40 °C and below 90 wt.% ethanol concentration, the measured flux and separation factor values were 0.126 kg/m^2^·h and 43.6, respectively. These results indicated that the M2.5 membrane outperformed the bare PVA membrane, confirming the enhanced performance achieved through the incorporation of silver nanoparticles [70].

Even if cross-linking provides chemical stability to the resulting membranes, the challenge lies in the fact that traditional chemical cross-linking methods often result in a reduction in hydroxyl groups in the PVA chain, leading to decreased membrane hydrophilicity, as well as compromised permeability and sometimes water selectivity. Therefore, it is crucial to explore alternative approaches that enable the retention of sufficient hydroxyl groups in cross-linked PVA membranes. Notably, Miranda et al. [71] and Bezuidenhout et al. [72] proposed novel methods for PVA cross-linking while preserving hydroxyl groups. Bezuidenhout et al., for instance, employed potassium persulfate, while Miranda et al. utilized ultraviolet light for self-cross-linking, enabling the retention of hydroxyl groups in the PVA chain. In a separate study, Gu et al. [73] fabricated cross-linked PVA membranes by employing ammonium persulfate (APS) for the purpose of separating a 95 wt.% ethanol–water system. The resulting membranes exhibited a remarkable performance, with a flux of 319.8 g/m^2^h and a separation factor of 3752. Consequently, the utilization of non-hydroxyl cross-linked PVA membranes for PV dehydration has garnered considerable attention among researchers. Likewise, Meisheng et al. [38] successfully developed water-selective PVA/PAN membranes using self-aggregation cross-linking reactions initiated by APS in the PVA matrix. The study extensively investigated the influence of PVA and APS contents on membrane properties, with particular focus on the impact of different physicochemical structures of PAN support. Furthermore, a 95 wt.% ethanol–water mixture was selected to evaluate the separation performance of the developed membranes. The experimental findings demonstrated favorable permeability–selectivity characteristics and long-term performance stabilities of the developed membrane. The PVA/PAN membrane exhibited good permeability–selectivity with a total flux of 117.6 g/m²h and a water content ratio of 99.99 wt.%.

### 2.2. Hydrophobic Polymers

As mentioned previously, the availability of hydrophobic membranes for PV is significantly limited due to the scarcity of suitable hydrophobic materials. Among the few hydrophobic materials that can be formed into appropriate pore structures for molecular separation are PDMS [74,75], poly(1-trimethylsilyl-1-propyne) (PTMSP) [76,77], poly(ether-block-amide) (PEBA) [57], and poly(octhylmethylsiloxane) (POMS) [78], which have been studied by the research community for separating ethanol molecules diluted in water systems (ethanol–water mixtures). For example, Chan et al. [79] fabricated membranes based on poly(ether block amide) (Peba 2533) with the incorporation of two organic fillers, namely 4-(trifluoromethyl)-N-(pyridine-2-yl)benzamide and 4-(dimethylamino)-N-(pyridine-2-yl)benzamide. The PV performance of these membranes was evaluated using an ethanol–water mixture containing 5 wt.% ethanol at temperatures ranging from 30 °C to 60 °C. The inclusion of the organic fillers resulted in improved pervaporation performance of the PEBA membrane, enhancing both permeate flux and separation factor when extracting ethanol molecules. In a more updated review, Serna-Vazquez et al. [80] have detailed revealed the advances of organophilic PV recovery of ethanol.

Specific polymer membranes, such as PDMS, are commonly utilized due to their wide availability, low cost, and simple processing [74,75]. However, these membranes often face a trade-off between permeability and selectivity. Among the various methods available for modifying surface properties, such as hydrophilicity/hydrophobicity and adhesion, plasma treatment has proven to be highly effective. For example, Johansson et al. [81] investigated the use of oxidized plasma (O_2_, CO_2_, H_2_O) to modify polycarbonate (PC) membranes and explored the associated etching mechanism. Mei-Sheng et al. [82] successfully developed a novel hydrophobic membrane through plasma polymerization of a polyether sulfone (PES) membrane, demonstrating favorable performance.

So far, hydrophobic PV membranes have mainly employed MFI, a hydrophobic zeolite, in their fabrication. This has been achieved through two predominant methods: (1) deposition of a thin film of MFI zeolite on a porous support [83], and (2) dispersion of MFI crystals within a polymer matrix [27]. These approaches have demonstrated remarkable performance in terms of both high flux and separation factors. The following section gives more details on developments of using inorganic materials for PV membranes.

## 3. Inorganic Membranes

Inorganic PV membranes are commonly synthesized using crystalline microporous materials, including zeolites, metal-organic frameworks (MOFs), and covalent organic frameworks (COFs). The most common inorganic materials used for PV dehydration of ethanol are shown in Table 2. These inorganic membranes offer several advantages over polymeric membranes, such as higher separation performance and improved stability, owing to their well-defined and rigid pore structures. However, the fabrication of defect-free inorganic membranes, primarily relying on hydrothermal synthesis, presents more challenges compared to the relatively straightforward production of polymeric membranes.

Zeolites, as the pioneering and most extensively researched family of inorganic materials for PV, possess tunable hydrophilic properties and sub-nanometer-sized pores that enable efficient molecular separations through strong preferential adsorption, rapid, and selective diffusion within the intergrown crystalline membrane. Among the various types of zeolites, hydrophilic zeolites, including NaA, CHA, and T-type, are commonly employed for organic dehydration applications, while a limited number of hydrophobic zeolites (like MFI) are suitable for organic recovery (separation) purposes.

Another class of extensively investigated hydrophilic membranes is amorphous silica membranes, which show promising potential for high-temperature organic dehydration while exhibiting adequate hydrothermal stability. Crystalline membranes derived from organic framework materials, such as metal (MOFs) and covalent organic frameworks (COFs) possessing versatile pore structures and functional groups, are also well-suited for PV separations. However, the water stability issue of MOFs must be resolved, and the inherently large pore size of COFs needs to be reduced to meet the prerequisites for efficient PV separations.

### 3.1. Zeolite Membranes

Zeolites, which are significant microporous aluminosilicate crystals, serve as the primary constituents of inorganic membrane materials that are being utilized for PV separation applications [86]. The pore size of zeolites is governed by the TO4 tetrahedral unit framework, where T can either be P, Si, or Al. Various zeolites possess distinct framework rings, such as 8-membered (e.g., DDR, LTA, CHA), 10-membered (e.g., FAU, MFI), and 12-membered (such as, MOR), resulting in pore sizes that ranges between 0.38 to 0.74 nm [87]. The major impact on the hydrophilicity of zeolites is caused by the Si/Al ratio, with lower ratios indicating higher hydrophilicity but lower acid stability [88]. The exceptional separation performance of zeolite-based PV membranes for organic-organic or water–organic mixtures even at higher temperatures is mainly attributed to their uniform and well-defined pores, tunable hydrophilicity, and remarkable thermal stability [89].

In a study, Fatima et al. [90] successfully synthesized a low-cost and highly permeable NaA zeolite membrane via an in situ crystallization approach using the secondary growth method on a kaolinite support. The synthesis conditions, including temperature, time of crystallization, and water ratio, were systematically investigated to obtain the optimal membrane quality and thus performance. The resulting membrane exhibited an impressive permeation flux of 8.49 kg/m^2^ h while reporting a remarkable separation factor (ca. 10,900) for the dehydration of 90 wt.% ethanol (at 75 °C).

Lin et al. [91] successfully obtained TFC membranes with enhanced performance by incorporating a zeolite interlayer and employing a two-pass interfacial polymerization (IP) process. The developed SUZ-4-enhanced TFC membrane, demonstrated a flux of 3.18 ± 0.3 kg/m^2^·h) and a separation factor of 1056 ± 150 when applied to the PV of 90 wt.% ethanol dehydration at 60 °C [92]. These results highlighted the significant improvement in membrane performance achieved by the SUZ-4 zeolite interlayer. The combination of the SUZ-4 zeolite interlayer and the two-pass IP process not only enhanced the membrane performance but also ensured the long-term stability of the membranes, making them a promising solution for efficient and reliable PV processes.

Han et al. [92] successfully fabricated an ultrathin zeolite X film with a total thickness of approximately 1 μm by subjecting seed monolayers to hydrothermal treatment in clear synthesis solutions. The performance of the membrane was evaluated for the dehydration of a 90/10 wt.% ethanol–water mixture at 65 °C. To some extent, the membranes demonstrated a remarkable total flux of 3.37 ± 0.08 kg/m^2^·h and a high separation factor of 296 ± 4 [93]. These results highlight the membrane’s ability to efficiently separate water from ethanol. Additionally, the membranes exhibited excellent stability over 5 h of PV operation, indicating their reliability and potential for long-term applications [92].

Guo et al. introduced, for the first time, the utilization of NaP1 zeolite in membrane pervaporation [93]. Structurally, NaP1 zeolite possesses a GIS type topology characterized by an 8-member ring structure, resulting in a pore limiting diameter of 3.0 Å. This particular pore size is highly suitable for the separation of water from alcohols, making it an attractive material [93]. To fabricate NaP1 zeolite membranes, seeded growth techniques were employed with varying Si/Al ratios of 1.9, 3.3, and 3.9 [93]. Subsequently, PV tests were conducted using an aqueous feed solution comprising 90 wt.% ethanol or isopropanol (at 348 K). Remarkably, the sample, recording a Si/Al ratio of 3, exhibited outstanding separation factors, surpassing those achieved by most existing zeolite membranes. Specifically, the separation factors for water–ethanol and water–isopropanol were reported as 200,000 and 36,000, respectively [93]. These findings confirmed the exceptional separation performance of the NaP1 zeolite membranes, underscoring their potential for efficient water-alcohol separation through this membrane-based technique.

### 3.2. Silica Membranes

The PV separation potential of microporous silica membranes was first explored three decades ago [94]. Several commercialized silica-based membranes have been developed for solvent dehydration, including ECN [95,96], HybSi^®^ [97], Pervatech [98], and Pervap SMS [99]. These membranes feature thin selective layers (less than 500 nm) of organosilica with exceptional hydrothermal stability and tunable porous structures.

Silica membranes have predominantly found application in organic dehydration processes due to their hydrophilic nature and the presence of sub-nanosized pores. The sol-gel method is commonly used to prepare silica membranes, which are typically deposited on a porous substrate to achieve high flux and mechanical strength. To achieve selective and efficient permeation during PV, the interconnectedness of sub-nanosized and nano-sized pores is crucial in silica membranes [100]. The pore structures can be modified by adjusting synthetic conditions, such as the type and concentration of precursors, catalysts, solvents, and the precursor-to-water ratio. However, pure silica membranes were found to be unstable under hydrothermal conditions [15]. Ma et al. [101] synthesized microporous silica membranes via sol–gel processing for PV separation of water–ethanol mixtures. The membranes were strategically supported on porous-alumina tubes (porosity: 50%, average pore size: 1 m, outer diameter: 11 mm, length: 70 mm). Coating the supports with fine-alumina particles (average diameter: 0.2–1.9 m) reduced the pore size before applying silica sol solutions. For testing, a 94 wt.% ethanol–water mixture was used. Initially, the permeate flux and separation factor decreased gradually during the separation process. After 6–10 h, the PV performance stabilized. The water flux ranged from 0.3 to 0.8 kg/m^2^h while the separation factor ranged from 10 to 500. During repeated PV experiments, water and ethanol flux were high initially and then declined, particularly at higher temperatures. This trend could be attributed to gradual physical and physicochemical adsorption, leading to pore plugging and reduced permeation.

In recent years, organic-inorganic hybrid silica membranes have emerged as a new generation of silica membranes, offering both high separation performance and stability. Remarkable PV dehydration performance and superior hydrothermal stability have been achieved using BTESE hybrid membranes [102]. In a study, Ravi et al. [103] synthesized functionalized silica (SBAPTS)–NSBC hybrid membranes using a sol–gel method followed by cross-linking, with the aim of facilitating PV separation of water–ethanol mixtures. To investigate the impact of membrane structure on PV performance, they systematically optimized the membrane composition and cross-linking density. Among the hybrid membranes tested, the most promising one (CPS-a) exhibited impressive results, with a permeation flux of 0.59 kg/m^2^h and a remarkable selectivity of 5282 for the dehydration of ethanol (90 wt.% ethanol) at 30 °C. These outcomes showcase the potential of the CPS-a hybrid membrane for efficient ethanol dehydration applications using PV.

The separation performance of silica membranes generally lies between that of polymeric and zeolite membranes [100]. This can be attributed to the inherent pore structures of silica membranes produced through the sol-gel process, which are more rigid and highly porous than polymers but lack the uniformity and high interconnectedness found in zeolites. Organic-inorganic hybrid silica membranes, known for their exceptional acid and hydrothermal stability, hold the potential to compete with other membranes for organic dehydration under harsh conditions, including high water content, temperature, low pH [16]. However, achieving precise control over pore size in silica membranes poses a significant challenge compared to zeolite membranes. Thus, further research should focus on molecular design strategies for silica sol and optimizing sintering conditions of the silica gel layer.

## 4. Mixed-Matrix Membranes

Mixed-matrix membranes (MMMs) have garnered significant attention in the field of membrane technology since the 1990s, primarily due to their ability to exceed the permeability–selectivity upper-bound tradeoff of polymeric membranes by incorporating high-performing fillers into the polymer matrix [13,104]. Unlike inorganic membranes, the synthesis techniques of MMMs follow the same general methods used for the fabrication of polymeric membranes, offering various advantages such as cost-effectiveness and scalability [105].

The molecular transport mechanism in MMMs can be described using the solution-diffusion model. The improved transport properties of polymer-based membranes caused by the incorporation of fillers are generally attributed to the improvement in diffusion and/or adsorption coefficients, as well as selectivity [13]. The use of MMMs for PV processes either enhances their preferential adsorption for organic molecules or water depending on the selection and incorporation of hydrophobic or hydrophilic filler into the polymeric matrix [105]. The presence of transport channels in fillers, whether permeable and/or selective, promotes preferential diffusion through the membrane. The effectiveness of this process heavily relies on the homogeneous distribution of fillers into the polymeric matrix, without causing any interfacial voids.

The physicochemical properties of the fillers have a crucial role in determining the separation performance of MMMs. Consequently, the development of MMMs has progressed in tandem with advancements in nanomaterials that can serve as fillers [63]. The first generation of MMMs, predominantly employed purely inorganic fillers such as zeolites and silica; however, achieving uniform filler dispersion and suppressing interfacial voids presented significant challenges, leading to suboptimal performance in these MMMs [13].

The emergence of novel nanomaterials, such as MOFs [106] and two-dimensional (2D) materials [107], has driven the development of second-generation mixed matrix membranes since the 2010s. These nanomaterials possess diverse functionalities and pore structures, making them highly compatible with polymers resulting in superior interfacial morphology and enhanced dispersion in MMMs [13].

### 4.1. Zeolite MMMs

The introduction of hydrophobic MFI zeolites into PDMS membranes has been recognized as a pioneering approach in mixed-matrix membranes (MMMs) for PV separation [83,86]. This development has contributed to better flux and enhanced ethanol–water separation factor. The improved performance can be credited to the hydrophobic nature of MFI zeolite fillers, which feature precisely engineered transport channels that promote the selective sorption and diffusion of ethanol relative to water. The separation performance of MFI/PDMS MMMs was found to be influenced significantly by three essential parameters: particle size, uniform particle dispersion, and zeolite loading [83].

To improve its separation features, zeolite surface modification has been confirmed to be favorable for attaining consistent filler dispersion at elevated loadings and, hence, results in improved zeolite dispersion within the polymeric matrix [108]. One way to attain zeolite surface modification is by utilizing silane coupling agents through the attachment of organic linkages, forming covalent bonds [87] or robust molecular interactions [83] with PDMS chains. For instance, PDMS MMMs demonstrated remarkable ethanol–water separation factors, reaching up to 59, with a silicalite-1 zeolite loading of up to 77 wt.% [25]. Zeolite loading was found to have a gradual effect on the separation factor, with the hydrophobic silicalite-1 zeolitic pores facilitating the permeation of ethanol while inhibiting water transport across the membrane. Nonetheless, earlier PDMS-based MMMs exhibited significant thickness, frequently reaching up to 100 μm, leading to low permeation fluxes, e.g., lower than 0.2 kg/m^2^h for 5 wt.% ethanol–water at 50 °C, thereby impeding practical utility.

To produce thinner MMM layers that would result in enhanced ethanol permeation while ensuring selectivity, it is essential to employ smaller-sized fillers. Another way to control the thickness of MMMs is by adjusting the fabrication conditions. In the case of PDMS MMMs, decreasing the viscosity of the casting solution results in thinner membrane layers, which, in turn, may also elevate the risk of filler sedimentation because of the greater density difference between the casting solution and filler. Recent research [109] findings revealed that by casting a PDMS solution filled with 67 wt.% of vinyltriethoxysilane-modified silicalite-1, featuring particle size < 500 nm, and controlling the viscosity through PDMS pre-polymerization, MMMs as thin as 5 μm were obtained. The resulting thin-film silicalite-1/PDMS MMMs showcased a favorable separation factor of 15.5 and an impressive flux of 5.52 kg/m^2^h for 5 wt.% ethanol–water mixtures at 50 °C.

Tanaka et al. [110] successfully fabricated thin LTA zeolite membranes using metal alkoxides. The choice of thin zeolite membranes was driven by the preference for PV dehydration, aiming to reduce the permeation resistance of water within the membrane. To investigate the impact of crystal growth conditions on separation performance, PV experiments were conducted using an ethanol–water mixture. Through optimization of the aging time of the secondary growth solution and secondary growth time, the thickness of the LTA zeolite membrane was reduced to less than 3 μm. Notably, for a 90 wt.% ethanol solution at 343 K, the membrane demonstrated a remarkable permeation flux of 6.3 kg/m^2^ h and an impressive separation factor exceeding 10,000. These findings highlight the potential of thin LTA zeolite membranes for highly efficient separation in ethanol dehydration.

Researchers have extensively explored hydrophilic MMMs for PV employing zeolites and polymers. These membranes not only exhibit improved preferential adsorption properties compared with hydrophobic MMMs, but also introduce additional molecular sieving properties due to the smaller pore sizes of the zeolites compared to organic molecules. The most common types of commercial hydrophilic zeolites with varying pore sizes that have been utilized for the synthesis of hydrophilic MMMs include type-A zeolites (such as 3A, 4A, and 5A, besides 13X). Generally, adding hydrophilic zeolites with large pore sizes has a direct impact on the permeation flux, while zeolite fillers with smaller sizes of pores tend to increase the separation factors during PV dehydration of organic compounds. For example, incorporating 20 wt.% of 5A zeolite into P84 polyimide resulted in a lower sorption capacity but higher water–isopropanol sorption selectivity compared with P84 filled with 20 wt.% of 13X zeolite. Additionally, introducing zeolites with the same pore size but higher hydrophilicity, such as NaX instead of NaY, enhanced water selectivity while simultaneously improving the permeances for water and ethanol in PVA membranes [111]. The versatility of hydrophilic MMMs with zeolites open up possibilities for tailoring membranes with improved selectivity and permeance for specific dehydration applications.

To enhance the performance of zeolite-based MMMs with better separation factors, further efforts should be focused on effectively reducing membrane thickness. Moreover, future focus could be directed towards developing innovative synthesis methods for zeolite nanoparticles and for establishing favorable interactions with the polymer matrix. By addressing these challenges and pursuing innovative solutions, the potential for advanced zeolite–polymer MMMs in PV applications can be further unlocked, bringing about significant advancements in membrane-based separation processes.

### 4.2. Silica MMMs

Silica nanoparticles have emerged as a common filler for the manufacture of polymer nanocomposites, which exhibit significantly improved bulk properties [102]. Building upon this concept, researchers have incorporated silica nanoparticles as fillers in polymer membranes to modulate their physical and/or chemical structures. Silica fillers play a dual role in MMMs, primarily serving to enhance hydrophilicity and to regulate chain conformation [94]. Typically, the sol–gel synthesis method enables the facile production of silica nanoparticles in aqueous polymer solutions. As a result, silica possesses a unique characteristic for MMMs, in that it can be formed in situ within the polymer matrix. This feature offers a promising solution for inhibiting filler agglomeration and interfacial voids [96]. Additionally, owing to its inherent hydrophilicity, silica has been incorporated into hydrophilic polymers to develop MMMs suitable for PV dehydration.

### 4.3. MOF MMMs

**Figure 3:**
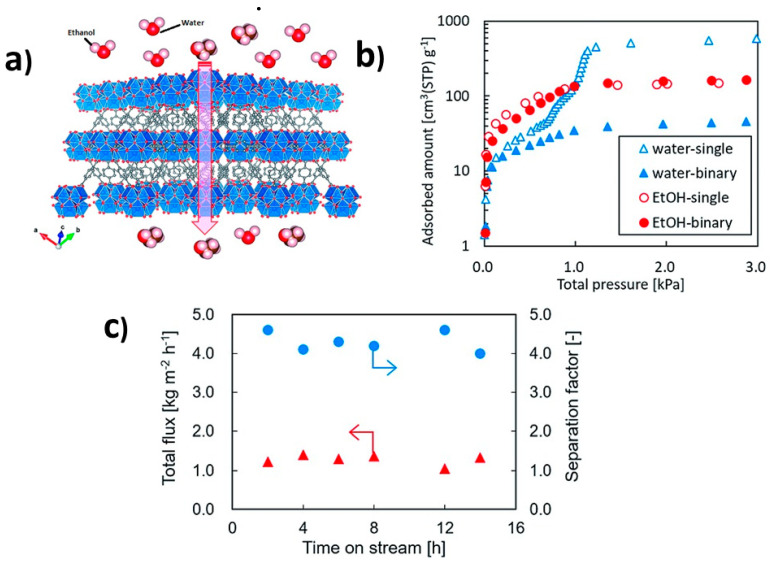
Schematic illustrations of the application of pure MOF-based membranes in pervaporation for the recovery of ethanol from aqueous solutions, (**a**) hypothetical mechanism of transport, (**b**) water and ethanol adsorption capacity, and (**c**) pervaporation separation performance (ethanol–water (10:90 wt.%) at 323 K). Adapted from [115].

MOFs have emerged as a new class of crystalline porous fillers for MMM fabrication. In comparison with zeolites, MOF fillers offer several advantages, including a superior compatibility with polymers because of the diverse pore structures, the presence of organic linkers, and the ability to achieve smaller particle sizes through gentle synthesis conditions. In recent years, a large number of MOFs have been synthesized; however, only a limited selection has been utilized as fillers in MMMs for PV applications. The notable ones in the list are ZIF-8, ZIF-7, and ZIF-71, besides UiO-66, Cu3(BTC)2/PDMS [112], HKUST-1/PVA [113], and Co(HCOO)_2_/PEBA [114]. MOFs themselves provide an extraordinary separation performance thanks to their preferential adsorption towards organics compared with water. This is the case for UiO-66, which displays slightly facilitated ethanol transport over its structure compared with water, as hypothetically reported in Figure 3a, which was further confirmed by adsorption measurements (Figure 3b). In Figure 3b, the term single refers to a single component such as water or ethanol, while the term binary refers to a solution (50/50) of ethanol and water. Experimentally, UiO-66 membranes tested for ethanol separation have reported outperforming permeation as high as 1.28 kg/m^2^ h, but a separation factor of 4.3 (see Figure 3c) [115]. In a recent study [116], Fang et al. proposed an innovative approach to enhance the separation performance of MOF membranes for water and ethanol separation. The strategy involved incorporating the 2,5-thiophenedicarboxylic acid (TDC) linker into the MOF-303 structure, partially replacing the 3,5-pyrazoledicarboxylic acid (PDC) linker. The aim was to increase the aperture size of the microporous channels in the pristine MOF-303 membrane, thereby improving the mass flux. PV tests were conducted on the prepared membranes to evaluate their performance in separating 90 wt.% ethanol at 60 °C. Outperforming the unmodified MOF-303, the mixed-linker MOF-303(50/50) membrane demonstrated superior mass flux of 0.092 kg/m^2^ h and a water–ethanol separation factor as high as 8500.

So far, the primary reason for the limited application of PV-based MMMs has been the stability of MOFs, particularly in the presence of liquids (including water). Assuming MOFs exhibit stability in liquid environments, two criteria are considered when selecting MOFs for the development of pervaporation MMMs: affinity and pore size. These criteria parallel the selection process employed for crystalline membranes. Hydrophilicity is often a determining factor when selecting a suitable MOF filler for hydrophobic or hydrophilic MMMs. Nevertheless, the interaction between pore-size-enhanced diffusion and affinity-enhanced sorption can introduce complications in PV performance, resulting in varied outcomes. For example, a hydrophobic MOF (such as ZIF-8) can be integrated into either a hydrophobic polymer (e.g., PDMS) or into a hydrophilic polymer (e.g., PVA) to attain enhanced separation performance in the resulting MMMs.

ZIF-8 has garnered significant attention as a PV membrane filler and stands as one of the most extensively studied MOF fillers. Table 3 enlists some examples of embedding MOF into polymer matrices to fabricate MMMs. In a research study, Pan et al. [117] crafted a superhydrophobic ZIF-8/PDMS/PVDF hybrid membrane, incorporating a nano-level bud-like ZIF-8 layer grown on a PDMS membrane through ZIF-8 particle dip-casting, secondary seeded growth, and hydrophobic modification using n-octadecylphosphonic acid for ethanol–water separation. The resulting optimal sandwich-like hybrid membrane exhibited an impressive separation factor of 17.4 and a total flux of 0.64 kg/m^2^ h with 5 wt.% of ethanol aqueous solution (at 30 °C). The approach of constructing a superhydrophobic inorganic layer on the PDMS membrane is a promising method for preparing sandwich-like hybrid membranes. For enhanced PV performance in MMMs, Zhu et al. [118] modified the surface of GO to enhance its surface hydrophobicity. In their research work, Zhu et al. reported the in situ growth of ZIF-8 particles on the GO surface, leading to the creation of ZIF-8@GO/PDMS MMMs. These MMMs demonstrated a separation factor of 22.2 and a permeation flux of 0.444 kg/m^2^ h for ethanol–water separation. According to the authors, the superior PV performance was attributed to the synergistic effect of GO nanosheets acting as a strong barrier and hydrophobic ZIF-8 nanoparticles with continuous inner channels.

Utilizing glycidyloxypropyltrimethoxysilane (TMS), Wang et al. [119] successfully synthesized UiO-66-MOF-based MMMs. The resulting UiO-66-TMS/PDMS MMMs displayed robust membranes with enhanced mechanical stability compared with the pristine UiO-66-NH_2_ MMMs. Remarkably, UiO-66-TMS/PDMS MMMs achieved a noTable 3.6-fold improvement in flux (at 50 wt.% loading) while maintaining selectivity, in comparison with PDMS pristine membranes during the 5 wt.% containing ethanol solutions. Concurrently, Lai et al. [120] investigated the use of (DUT-5) MOF-based MMMs for ethanol dehydration. The resultant MMMs revealed superior ethanol permeability through pervaporation compared with the pristine membrane. In a study [121], ZIF-7 microparticles with sizes ranging from 1 to 2 μm were successfully incorporated into chitosan (CS) polymer for the separation of water–ethanol mixtures using a pervaporation set-up. The resulting MMMs with 5 wt.% ZIF-7 loading exhibited a separation efficiency 19 times higher than that of pure CS membranes, albeit with a lower flux due to the rigidified cross-linking between the zinc atoms within ZIF-7 and the –NH_2_ groups of the CS polymer. Additionally, considering the high hydrophilicity of HKUST-1, Coronas et al. [122] and his team prepared polyimide-based MMMs for the PV separation of water–ethanol mixtures by incorporating HKUST-1 particles at 20–40 wt.% loading into commercial polyimide Matrimid^®^ 5218 [122]. The water flux increased from 240 g/m^2^h for the bare polyimide membrane to 430 g/m^2^h for the 40 wt.% Cu_3_(BTC)_2_-based MMM, while the separation factor (>200) remained relatively unchanged. In the pursuit to enhance water permselectivity, Huayan incorporated hydrophilic zirconium-based NU-906 nanoparticles into CS, resulting in NU-906/CS MMMs for alcohol dehydration. The introduction of NU-906 conferred an excellent water affinity and structural stability to the hybrid membranes. The incorporation of NU-906, with its hydrophilicity and ordered porosity, led to significantly improved water selectivity and permeability in the hybrid membranes. The optimal membrane with 5 wt.% NU-906 exhibited an impressive flux of 1086 g/m^2^h and an outstanding separation factor of 2651 for 90 wt.% ethanol dehydration at 76 °C.

**Table 3:** MOF-based MMMs for ethanol dehydration via PV.

Polymer	MOF	Feed Composition (EtOH/H_2_O)	T °C	Flux (g/m^2^h)	Separation Factor	Selectivity	Reference
CS	ZIF-7	90/10	25	322	2812	N/A	[121]
CS	Al-MOF	90/10	25	458	2741	N/A	[123]
CS	DUT-5	90/10	25	378	3429	N/A	[124]
CS	MOF-801	90/10	70	1937	2156	2641.14	[125]
PVA	ZIF-8	80/20	25	486	4725	N/A	[126]
PVA	Zr-MOF	90/10	30	46.3	46.3	64.63	[127]
SA	ZIF-8	90/10	76	879	678	812.48	[128]
SA	EuBTB	90/10	76	1996	1160	1374..64	[129]

When compared with zeolite MMMs, the best part of MOF MMMs is that they offer unique advantages in terms of nanofiller synthesis, uniform filler dispersion within the polymer matrix without causing any interfacial voids, and the ability to achieve much thinner membrane layers. It is worth noting that while ZIF-8 has not been successful in the development of crystalline PV membranes, it has demonstrated an excellent pervaporation performance when used as a filler in hydrophobic polymeric membranes. Future research efforts should focus on addressing the long-term stability challenges associated with MOF-based MMMs, given the known water stability issues, as observed in ZIF-8 crystalline membranes. When exploring MOFs in MMMs, there is a need to identify water-stable MOFs and the appearance of suitable aperture sizes to broaden the range of promising MOF MMMs.

### 4.4. COF MMMs

The incorporation of COF fillers with preferential adsorption properties and fast diffusion channels can enhance the PV performance of polymeric membranes. To some extent, PV dehydration stands as a critical step in producing anhydrous ethanol, necessitating the utilization of water-selective membranes. Notably, researchers have been increasingly exploring the construction of various COF-based MMMs tailored for PV applications. By skillfully incorporating COFs into polymeric membranes, these MMMs effectively modulate the adsorption and diffusion of components during permeation vaporization, leading to impressive selectivity and permeability performances. For instance, Yang et al. [130] added COF SNW-1 into the sodium alginate (SA) matrix to prepare a COF-based hybrid membrane and used it for ethanol dehydration. SA has been used as a membrane material due to its good membrane forming property and desirable separation performance. Nevertheless, there are certain limitations in the permeability and selectivity of pure SA membranes. To obtain a better performance, it is vital to further improve hydrophilicity. Herein, the enrichment of COF improved the hydrophilicity and water absorption ability of this membrane. This hybrid matrix membrane exhibited better thermal and mechanical stability, an excellent anti-expansion performance, and long-term running properties. The permeate flux of the hybrid membrane was 2170 g/m^2^ h with a separation factor of 2099 when 90 wt.% ethanol aqueous solution was pervaporated at 76 °C.

**Figure 4:**
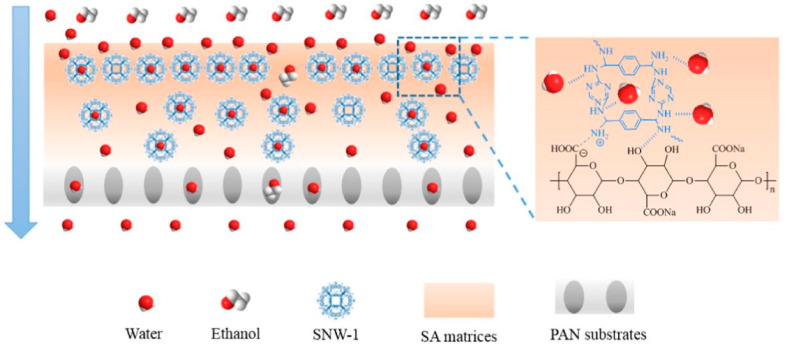
COF/SA MMM: schematic of the membrane structure and transport of water and ethanol [130].

By incorporating melamine-based SNW-1 into a sodium alginate (SA) matrix, a hybrid membrane, referred to as the SNW-1/SA hybrid membrane (see Figure 4), was successfully fabricated on a polyacrylonitrile substrate for the purpose of ethanol dehydration from an aqueous solution. This innovative approach resulted in an improved separation factor and permeance flux [130]. The enhanced permeance performance was attributed to the increased hydrophilicity and the presence of more water channels due to the embedded SNW-1 particles [131].

**Figure 5:**
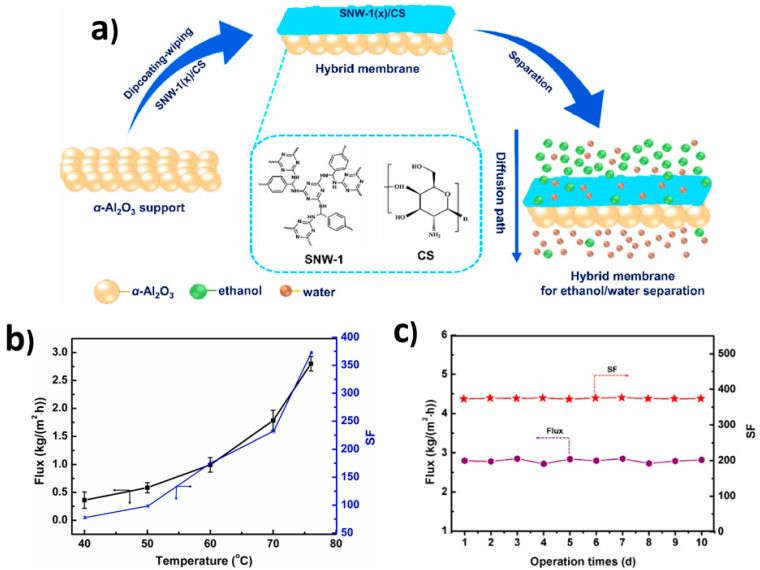
(**a**) Graphical depiction of SNW-1/Cs MMMs onto alumina support, (**b**) their separation performance for the dehydration of ethanol, and (**c**) long-term operation. Adapted from [132].

Drawing inspiration from the performance of melamine-based COF, Luo et al. [132] extended their work to develop an organic hybrid membrane. As illustrated in Figure 5a, melamine-based SNW-1 was integrated into a CS membrane using a straightforward dip-coating–wiping method. The addition of SNW-1 not only enhanced the hydrophilicity, but also improved the stability of the resulting SNW-1/CS melamine-based hybrid membrane. The prepared hybrid membrane exhibited excellent water-selective properties, achieving high flux and demonstrating remarkable long-term stability. It showed promise as a water-selective membrane for practical applications. To provide a comprehensive evaluation, a comparison experiment was conducted, and the SNW-1-incorporated SA hybrid membrane was also fabricated and assessed for its PV performance in the presence of 90 wt.% ethanol–water solution. The melamine-based SNW-1(10)/CS membrane achieved a superior performance with a separation factor of 373 and a flux of 2.8 kg/m^2^h (see Figure 5b). Apart from the effective separation performance, these MMMs showed a stable performance at 76 °C for a 10-day test with stable flux and separation factor (Figure 5c).

In a study, Dan et al. [133] developed novel asymmetric PV MMMs by integrating a low-density Schiff base network framework (SNW-1) into a relatively high-density poly(vinyl alcohol) (PVA) matrix. The incorporation of SNW-1 nanoparticles, featuring water-selective pore structures within the PVA body or on the membrane surfaces, significantly enhanced the membrane’s separation performance. Specifically, MMMs with just 1.5 wt.% SNW-1 demonstrated an impressive separation factor of 751 and a total flux of 254 g/m^2^h for 90 wt.% ethanol aqueous. Notably, the MMMs exhibited a remarkable long-term operating stability, maintaining their initial total flux and separation factor values even after 120 h of operation at 75 °C. These findings highlight the potential of COFs-based MMMs for applications in ethanol or other forms of alcohol dehydration.

The emerging COF fillers have demonstrated significant potential in both hydrophilic and organophilic membranes, enhancing the PV performance of polymeric membranes even at low loading levels. Given the limited molecular sieving effect of COFs’ large intrinsic pores, the specific mechanisms underlying the enhancement of transport properties by COF fillers remain unclear and are a current scope of research. In this regard, further advanced characterization of the physicochemical properties of COF MMMs would be beneficial for gaining a deeper understanding of the transport mechanisms responsible for their excellent PV performance.

## 5. Conclusions and Future Outlook

This review states the ongoing progress of the emerging membranes for the separation of ethanol from diluted or concentrated mixtures via PV. The current achievements in terms of membrane materials, structure design, fabrication approach, separation performance, and characteristics of ethanol separation from water have been thoroughly discussed. The molecular transport mechanism is compared from the perspective of membrane material and process. The major achievements alongside the most common challenges associated with each type of membrane material are summarized below:

*Polymeric membranes*: They continue to dominate the pervaporation field due to their low cost and scalability. Hydrophilic membranes, such as PVA, and hydrophobic membranes, such as PDMS, remain the benchmark materials for ethanol dehydration and ethanol separation from diluted systems, respectively. Despite their exceptional chemical and physical properties, the use of these pristine polymers for fabricating membranes is adversely affected due to their low permeabilities, which are far below the acceptable separation performance for versatile operating conditions. Recent efforts have focused on enhancing membrane structural stability, reducing membrane thickness, producing defect-free separation layers and minimizing the decline in membrane performance. Although there is a high demand for the separation of organic–organic mixtures, deploying current polymers on a large scale for the separation of such solutions is limited because of the performance issues of these polymers, including the lack of discrimination ability and structural stability of these polymers. Polyimides, because of their stiff and rigid chains, have been proven to offer excellent resistance to harsh operating conditions and aggressive solvents; however, they suffer low permeation flux. To expand their use for applications in the PV field, polyimides need to be transformed into thin-skinned asymmetric membranes.

*Inorganic membranes*: As characterized by their highly porous and relatively rigid structures, they offer higher permeance and good selectivity compared with polymeric membranes. Zeolite membranes, in particular, have been extensively studied and demonstrate a superior performance among PV membrane materials. The commercialization of zeolite membranes, such as NaA membranes, has achieved an excellent organic dehydration performance through the use of hollow fiber substrates and optimized seeding methods. The main drawback of NaA membranes is their acid instability, which has been mitigated by synthesizing CHA zeolite-based membranes, which possess higher Si/Al ratios. MFI zeolites, with their hydrophobic nature, exhibit outstanding selectivity for ethanol recovery because of their excellent pore size discrimination ability. The performance of MFI membranes can be significantly increased by growing zeolites into nanosheet-seeded or b-oriented layers. To obtain silica membranes with an enhanced hydrothermal stability, various approaches have been used, including the synthesis of membranes through organic–inorganic hybrid materials. Precise molecular design of the silica network is essential to enhance the selectivity for organic dehydration. Crystalline membranes developed either by utilizing MOFs or COFs are still in the early stages of development for PV applications, and have mostly been investigated at lab-scale. Among them, only the UiO-66 MOF membrane has shown excellent potential for application in organic–organic separation and organic dehydration because of its outstanding stability in water. If the pore size of some of the emerging COFs membranes are adjusted carefully either during or after synthesis, they can exhibit efficient separation of water from large-sized alcohols comparable to zeolite membranes.

*Mixed-matrix membranes (MMMs)*: These have been developed by integrating suitable fillers into appropriate polymers to blend their properties. The first generation of MMMs was developed by utilizing purely inorganic fillers, primarily zeolites or silica. Particularly, hydrophobic zeolite MMMs have achieved greater success in terms of enhanced performance compared with their counterpart hydrophilic zeolite MMMs, due to the ease of forming interfacial voids and chain rigidification with hydrophilic polymers compared with hydrophobic polymers. Although hydrophobic zeolite MMMs can achieve high separation factors at higher loadings, the flux remains unimpressive due to the thickness of the MMM layer required to ensure defect-free membranes. In contrast, MMMs developed by adding silica fillers have been found to have uniform filler dispersion and intact interfaces because of in situ filler formation. However, their potential to utilize transport channels remains limited due to the nonporous nature of silica. The second generation of MMMs, developed by utilizing either MOFs, COFs, or 2D materials, have overcome the pitfalls associated with nanoparticle synthesis, formation of interfacial voids, uniform filler dispersion, and membrane thickness, which is commonly encountered in zeolite MMMs [134,135,136]. Emerging COF fillers have been found to offer an excellent performance enhancement for both hydrophilic and hydrophobic polymers because of their versatile pore structures and crystalline polymer nature. As for 2D fillers, such as GO nanosheets, these have been deployed into polymeric membranes to obtain enhanced alcohol dehydration results. The specific functions of COF or 2D fillers in enhancing transport properties require further exploration, considering the multiple transport channels they introduce. For large-scale PV application of emerging materials, such as MOFs, COFs, 2D materials, and their MMMs, it is important to test these materials under rigorous operating conditions for long-term stability. Various issues associated with membrane surface morphology, such as filler pore blockage, chain rigidification, interfacial voids, and filler aggregation, need to be addressed, as they usually complicate the transport channels and, hence, hinder the overall separation performance of MMMs. Once these challenges are successfully addressed, thin, robust, and defect-free MMMs should be fabricated on large-scale PV applications.

## Figures and Tables

**Table 2:** Separation performance of inorganic membranes for ethanol–water dehydration.

Membrane	Feed (wt.% Ethanol)	T (°C)	Total Flux (kg/m^2^h)	Separation Factor	Ref.
ECN-Silica	89.7	70	2.330	60	[84]
Perv-Silica	89.0	70	2.000	160	[84]
Zeolite-A	89.9	70	1.120	18,000	[84]
Zeolite-T	89.9	70	0.910	1000	[84]
M-350	94.3	70	1.500	65	[85]
M-450	94.2	70	0.820	346	[85]
M-550	94.1	70	0.760	1675	[85]

## Data Availability

Data are contained within the article.

## Nomenclature

*A*	membrane area (m^2^)
*D*	diffusion coefficient (10^−8^ cm^2^/s)
*J*	permeation flux (g/m^2^h)
*l*	membrane thickness (μm)
*P*	permeability (g m/m^2^h kPa)
*P/l*	permeance (g/m^2^h kPa)
*S*	sorption coefficient (cm^3^(STP)/cm^3^ atm)
*x*	weight percent of components in the feed
*y*	weight percent of components in the permeate
***Greek Letter***	
*α*	selectivity
*β*	separation factor
*δ*	solubility parameter (MPa^1/2^)
***Abbreviations***	
2D	two-dimensional
BTESE	1,2-bis(triethoxysilyl)ethane
COF	covalent-organic framework
CS	chitosan
DMC	dimethyl carbonate
ECN	Energy Research Centre of the Netherlands
GO	graphene oxide
MMM	mixed-matrix membrane
MOF	metal-organic framework
MTBE	methanol/methyl *tert*-butyl ether
NF	nanofiltration
PA	polyamide
PAN	poly(acrylonitrile)
PBI	polybenzimidazole
PDC	3,5-pyrazoledicarboxylic acid
PDMS	polydimethylsiloxane
PE	polyether
PEBA	poly(ether-block-amide)
PEC	polyelectrolyte complex
PES	poly(ether sulfones)
PI	polyimide
PIMs	polymers of intrinsic microporosity
PMPS	polymethylphenysiloxane
POMS	polyoctylmethylsiloxane
POSS	polyhedral oligomeric silsesquioxanes
PTMSP	poly(1-trimethysilyl-1-propyne)
PU	polyurethane
PVA	polyvinyl alcohol
SA	sodium alginate
SNW-1	schiff base network framework
TDC	2,5-thiophenedicarboxylic acid
TEOS	tetraethylorthosilicate
TFC	thin-film composite
TMS	glycidyloxypropyltrimethoxysilane
